# Single-Cell Proteomics Decodes the Cellular Response to Lysosomal Storage in *C. elegans* Coelomocytes

**DOI:** 10.3390/ijms27104197

**Published:** 2026-05-08

**Authors:** Yiming Lei, Fanghua Lu, Qinqin Xu, Lishuan Wu, Qun Fang, Hongyun Tang

**Affiliations:** 1 School of Life Sciences, Fudan University, Shanghai 200433, China; 2 State Key Laboratory of Gene Expression, School of Life Sciences, Westlake University, Hangzhou 310030, China; 3 Westlake Laboratory of Life Sciences and Biomedicine, Hangzhou 310030, China; 4 Single-Cell Proteomics Research Center, and Zhejiang Key Laboratory of Intelligent Manufacturing for Functional Chemicals, ZJU-Hangzhou Global Scientific and Technological Innovation Center, Zhejiang University, Hangzhou 311200, China; 5 Department of Chemistry, Zhejiang University, Hangzhou 310058, China

**Keywords:** *Caenorhabditis elegans*, single-cell proteomics, lysosomal storage, cellular stress response, proteostasis

## Abstract

Lysosomal storage, characterized by the progressive accumulation of undigested substrates in the lysosomal lumen, is a primary driver of various lysosome-related diseases. However, single-cell proteomic remodeling during lysosomal storage remains elusive, and the cellular responses for coping with this condition are poorly understood. Here, we employed deep-coverage single-cell proteomics to analyze *C. elegans* scavenger cells (coelomocytes) undergoing lysosomal storage. Our analysis revealed profound proteomic remodeling characterized by the massive, asymmetric upregulation of nearly 1000 proteins. We identified a coordinated compensatory response involving the robust induction of endoplasmic reticulum (ER) quality control, including ER unfolded protein response and ER-associated degradation, systemic hyperactivation of the ubiquitin–proteasome system (UPS), and a discordant mitochondrial response featuring concurrent bioenergetic upregulation and severe proteostatic stress. Collectively, this single-cell analysis establishes a high-resolution molecular blueprint of the hierarchical strategies cells employ to survive lysosomal collapse via compensatory quality control mechanisms.

## 1. Introduction

Cellular homeostasis depends on a sophisticated network integrating proteostasis, organelle homeostasis, and metabolic homeostasis [1,2,3]. Central to this network is the lysosome, a degradative hub that orchestrates cellular responses to metabolic, proteotoxic, and organelle stress [4,5]. By managing the turnover of damaged organelles and protein aggregates, the lysosome is indispensable for maintaining intracellular integrity, a function of paramount importance in long-lived, post-mitotic cells [6,7].

Impaired lysosomal degradative capacity constitutes the fundamental pathogenic basis of lysosomal storage disorders (LSDs). Comprising over 70 distinct conditions, these devastating inherited metabolic diseases progress relentlessly, often affecting neurons and professional scavenger cells [8,9,10], and triggering a catastrophic cascade that extends far beyond mere substrate accumulation [11]. This raises a critical question: how do individual cells adapt and survive amidst such lysosomal failure? The comprehensive functional proteomic blueprint governing cellular responses to lysosomal decline, particularly at single-cell resolution, remains to be systematically elucidated.

To decode the proteomic landscape during lysosomal storage, we utilized coelomocytes, the professional scavenger cells of *C. elegans*. Biologically, coelomocytes continuously uptake the pseudocoelomic fluid via endocytosis, relying heavily on their robust lysosomal network to clear extracellular waste and maintain pseudocoelomic homeostasis [12]. Functionally analogous to mammalian macrophages, this prominent baseline activity makes them a suitable in vivo model to recapitulate the lysosomal burden experienced by human scavenger cells. By coupling the massive secretory output of the intestine with the high-volume endocytic capacity of the coelomocytes, a profound accumulation of a degradation-resistant protein within their lysosomal lumen is induced [13]. However, analyzing the proteomic response of these cells presents a significant technical challenge; their extreme scarcity, with only six coelomocytes per organism, precludes the use of conventional bulk proteomic workflows. To overcome this limitation, we integrated a high-precision, microfluidic-based Pick-up Single-cell Proteomic Analysis (PiSPA) platform [14] with ultra-sensitive mass spectrometry [15,16,17]. This strategy enabled, for the first time, the acquisition of high-fidelity, deep-coverage proteomic profiles from individual coelomocytes, thereby elucidating the cellular mechanisms employed to cope with lysosomal storage.

Here, we provide a comprehensive molecular map detailing the cellular response to lysosomal failure at single-cell resolution. By analyzing the global proteomic shifts, we aimed to uncover how lysosomal storage triggers compensatory stress responses across other major organelle systems, specifically focusing on the endoplasmic reticulum (ER), the ubiquitin–proteasome system, and mitochondria. Collectively, our findings provide a molecular blueprint of the compensatory strategies cells deploy to cope with the burden on their primary recycling center.

## 2. Results

### 2.1. Establishing a High-Precision Single-Cell Proteomics Workflow for C. elegans Coelomocytes

**Figure 1:**
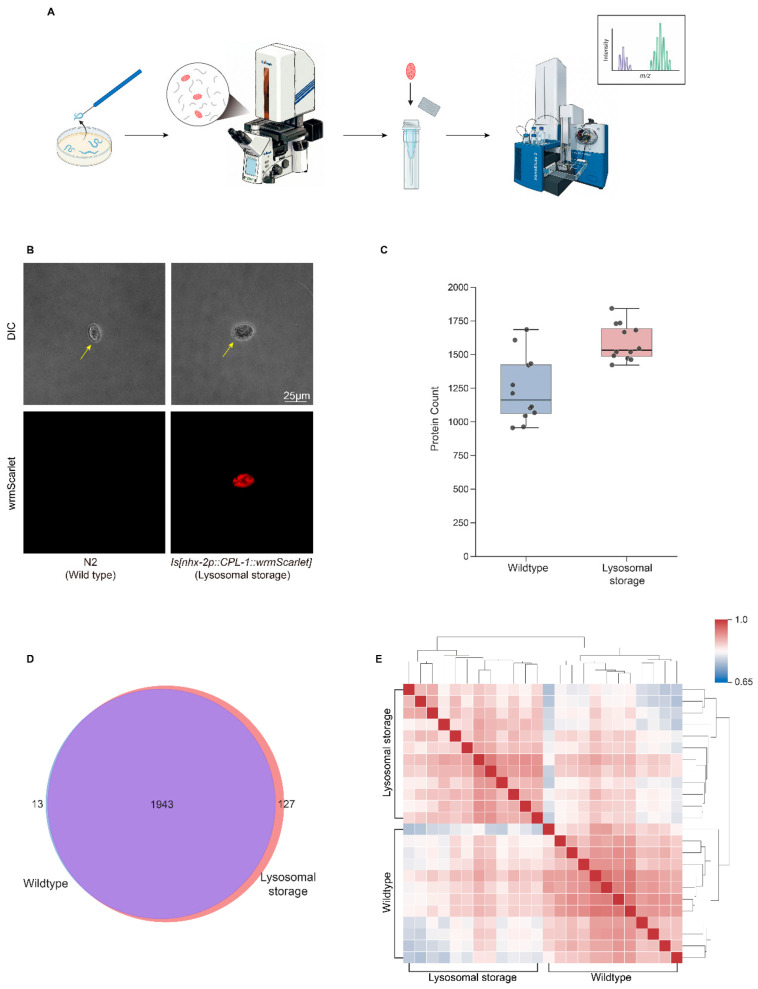
Visual-guided single-cell proteomic workflow and data quality assessment. (**A**) Schematic of the Pick-up Single-cell Proteomic Analysis (PiSPA) workflow for profiling coelomocytes. Target cells were isolated from dissected *C. elegans* using fluorescence-guided micropipetting, followed by automated processing and LC-MS/MS analysis. (Created with BioRender.com). (**B**) Representative DIC and fluorescence micrographs showing the identification of wild-type (N2) coelomocytes and CPL-1::wrmScarlet-positive storage model coelomocytes (indicated by yellow arrows) prior to capture. In the *jefIs41* transgenic strain, the intestine-specific promoter (*nhx-2p*) drives the expression of a fluorescently tagged lysosomal protease (CPL-1::wrmScarlet). This transgene product is secreted by the intestine and massively endocytosed by coelomocytes, thereby inducing lysosomal storage in these scavenger cells. Scale bar, 25 μm. (**C**) Box plots demonstrating proteomic depth across all samples (*n* = 12 cells per group). The plot shows the number of proteins quantified per single coelomocyte. (**D**) Venn diagram illustrating the overlap of protein identifications between the wild-type and lysosomal storage groups, showing a large shared proteome. (**E**) Pearson correlation heatmap of global protein expression. High intra-group correlation coefficients (r) confirm the high reproducibility of the single-cell proteomic workflow.

To resolve the cellular proteomic landscape during lysosomal storage at high resolution, we developed a visually guided workflow to isolate individual coelomocytes using the PiSPA platform (Figure 1A). Building on prior work, we utilized the *jefIs41[nhx-2p::CPL-1::wrmScarlet]* transgene, which drives the accumulation of endocytosed, intestine-secreted CPL-1 [human Cathepsin L]::wrmScarlet in coelomocytes, to induce lysosomal storage [13]. Coelomocytes were identified by their characteristic oval morphology and, in the storage model, by the distinct fluorescence of CPL-1::wrmScarlet within these cells (Figure 1B). We isolated 12 single coelomocytes each from wild-type (N2) and lysosomal storage model groups for single-cell proteomic analysis via mass spectrometry. Given that an adult *C. elegans* possesses a fixed complement of only six coelomocytes, this collection of 12 individual cells effectively represents two complete biological equivalents of the entire coelomocyte population, providing a representative assessment of this rare cell type. Following stringent quality control, our analysis achieved exceptional proteomic depth at the single-cell level, quantifying an average of 1416 proteins per coelomocyte, with individual counts ranging from 956 to 1843 across the 24 cells analyzed (Figure 1C; Supplementary Table S1). Notably, coelomocytes from the storage model exhibited significantly greater proteomic depth (~1591 proteins) compared to the wild-type group (~1240 proteins). This increase likely stems from lysosomal storage pathology, specifically the accumulation of undegraded substrates and a broad compensatory response to lysosomal dysfunction.

Supporting the consistency and reliability of the identified proteome, a Venn diagram revealed a core set of 1943 proteins shared between both groups, with only 13 and 127 proteins exclusively detected in the wild-type and lysosomal storage groups, respectively (Figure 1D), thereby providing a stable baseline for comparison. Furthermore, Pearson correlation analysis of global protein expression profiles demonstrated robust reproducibility among biological replicates within each group (mean Pearson’s r ≈ 0.89 for N2 and 0.88 for the storage group; minimum intra-group r > 0.82), as well as distinct clustering between the two groups (Figure 1E). Collectively, these rigorous quality control metrics confirm the high reliability and deep coverage of our single-cell proteomic dataset, establishing a solid foundation to investigate the subcellular stress landscape triggered by lysosomal storage.

### 2.2. Lysosomal Storage Induces Profound and Systemic Proteomic Remodeling

**Figure 2:**
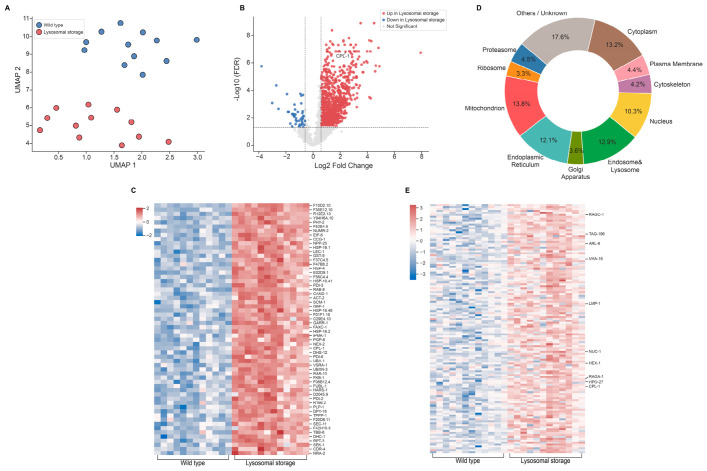
Lysosomal storage triggers profound and systemic proteomic remodeling. (**A**) UMAP plot of single-coelomocyte proteomes. Each dot represents the proteome of a single cell, colored by group [wildtype N2 or lysosomal storage (*jefIs41[nhx-2p::CPL-1::wrmScarlet]*)]. The complete segregation of the two groups indicates a massive, systemic proteomic shift. (**B**) Volcano plot displaying differentially expressed proteins (DEPs) between the lysosomal storage (*jefIs41[nhx-2p::CPL-1::wrmScarlet]*) and N2 groups. Significantly up-regulated (red) and down-regulated (blue) proteins are highlighted (FDR < 0.05; |Fold Change| ≥ 1.5). The storage-inducing protein, CPL-1::wrmScarlet, is noted as a positive control. (**C**) Hierarchical clustering heatmap of the top 60 most significant DEPs. Color intensity represents the Z-score normalized protein abundance across individual cells. (**D**) Donut chart showing the subcellular distribution of all 999 up-regulated DEPs in the coelomocytes of *jefIs41[nhx-2p::CPL-1::wrmScarlet]* worms based on UniProtKB annotations. Percentages indicate the proportion of altered proteins in each compartment, revealing a multi-organelle response. (**E**) Targeted single-cell heatmap of the 129 significantly upregulated endo-lysosomal DEPs. The lysosomal proteins, spanning structural components, trafficking/fission regulators, proton pumps, hydrolases, and sensing machineries, are indicated on the *y*-axis. Color intensity represents the Z-score normalized protein abundance across individual coelomocytes.

Uniform Manifold Approximation and Projection (UMAP) of single-coelomocyte proteomes revealed complete spatial segregation between wild-type and storage groups (Figure 2A), indicating profound, systemic remodeling of the cellular proteome. Subsequent quantitative differential analysis delineated the scope of this reorganization, uncovering a striking asymmetry in protein regulation. Applying stringent thresholds (FDR < 0.05 and |Fold Change| ≥ 1.5), we identified 999 significantly upregulated proteins compared to only 48 downregulated proteins (Figure 2B; Supplementary Table S2). This near-unidirectional shift supports a model of impaired protein clearance coupled with a massive compensatory cascade. As an internal control, CPL-1, the protein fused to wrmScarlet to induce storage [13], ranked among the most highly upregulated targets. Hierarchical clustering of the 60 most significantly altered proteins, all of which were upregulated, further underscored this consistent divergence (Figure 2C).

Furthermore, mapping the subcellular localization of the 999 upregulated proteins using the UniProtKB database revealed that alterations were not confined to a single pathway but spanned nearly every major subcellular compartment (Figure 2D), indicating that lysosomal storage triggers a multi-organelle stress response.

Notably, within this global response, 129 of the 999 upregulated DEPs were specifically categorized as endosomal and lysosomal proteins. To explicitly validate the occurrence of lysosomal storage and characterize the local compensatory machinery, we performed a targeted analysis of these 129 endo-lysosomal DEGs (Figure 2E). This targeted single-cell heatmap highlighted an expansion of the lysosomal proteins across all individual storage coelomocytes. We observed a robust accumulation of lysosomal proteins encompassing all core functions, including structural membrane scaffolds (LMP-1 [human LAMP1]), vesicular trafficking and fission regulators (ARL-8 [human ARL8A], HPO-27 [human MROH1]), V-ATPase proton pump subunits (VHA-16 [human ATP6V0D1]), luminal hydrolases (NUC-1 [human DNASE2], HEX-1 [human HEXA/B], TAG-196 [human CTSF]), and nutrient-sensing components (RAGC-1 [human RRAGC/D], RAGA-1 [human RRAGA/B]). While the massive accumulation of the endocytosed CPL-1::wrmScarlet drives the primary storage burden, the concomitant, broad upregulation of these endogenous lysosomal markers supports a concerted, adaptive biogenesis of the endo-lysosomal compartment to cope with the undigested substrate.

### 2.3. An Induction of Endoplasmic Reticulum Quality Control Responses During Lysosomal Storage

**Figure 3:**
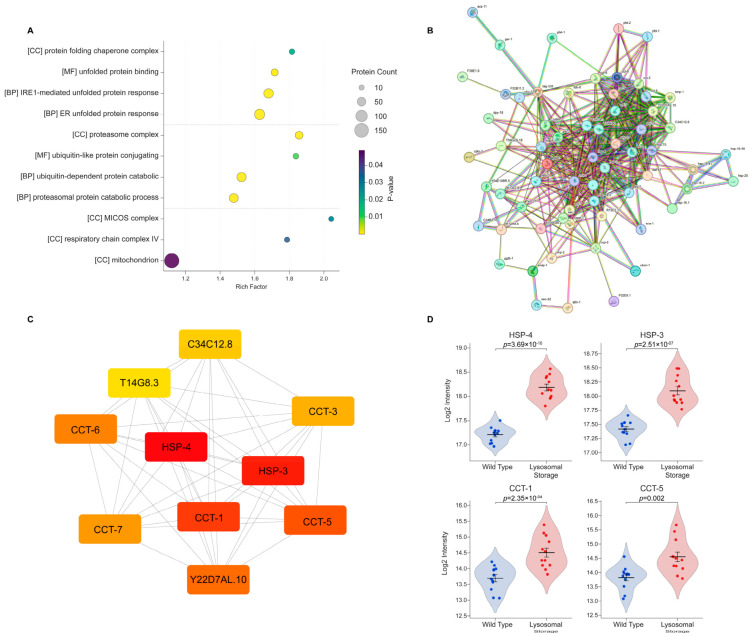
Functional enrichment analysis reveals the induction of ER quality control processes during lysosomal storage. (**A**) GO enrichment analysis of upregulated proteins in the coelomocytes of *jefIs41[nhx-2p::CPL-1::wrmScarlet]* worms. The bubble plot displays significant terms from Biological Process (BP), Molecular Function (MF), and Cellular Component (CC). Bubble size corresponds to the number of genes, the *x*-axis represents the enrichment factor, and color indicates statistical significance (*p* < 0.05). (**B**) Protein–protein interaction (PPI) network of upregulated proteins in the coelomocytes of *jefIs41[nhx-2p::CPL-1::wrmScarlet]* worms associated with the ER unfolded protein response, generated using STRING. (**C**) The top 10 hub proteins from the chaperone PPI network, identified by the Maximal Clique Centrality (MCC) algorithm, in the lysosomal storage condition. Node color intensity (yellow to red) correlates with a higher MCC score, indicating greater network centrality. (**D**) Violin plots showing the single-cell abundance of key ER stress regulators: HSP-4, HSP-3, CCT-1, and CCT-5. Each dot represents a single cell. Horizontal lines indicate the mean ± SEM. Statistical significance was determined using a two-tailed Student’s *t*-test.

To deconstruct this systemic response, we performed Gene Ontology (GO) enrichment analysis on upregulated proteins (Figure 3A; Supplementary Table S3). The analysis revealed distinct organelle-specific responses, with the most significant GO terms converging on three major functional hubs: the endoplasmic reticulum (ER) unfolded protein response (UPR^ER^), the ubiquitin–proteasome system (UPS), and mitochondrial function. We first examined the ER stress network by constructing a protein-protein interaction (PPI) network of upregulated ER-associated proteins, which revealed a highly interconnected chaperone response (Figure 3B). Application of the Maximal Clique Centrality (MCC) algorithm identified the top 10 hub proteins (Figure 3C). This core network consisted primarily of major molecular chaperones, including the canonical ER chaperone BiP (HSP-3/-4) and essential components of the CCT/TRiC cytosolic chaperonin complex [18,19], indicating a coordinated folding response.

Single-cell quantitative profiling using violin plots further confirmed the induction of these regulators (Figure 3D). The substantial upregulation of HSP-4, the *C. elegans* homolog of the master UPR regulator BiP/GRP78 [20], alongside its paralog HSP-3, provides clear evidence of transcriptional UPR activation. Concurrent up-regulation of HYOU1/GRP170 (T14G8.3), an essential nucleotide exchange factor for HSP-4, and cytosolic chaperonins such as CCT-1 and CCT-5 [human TRiC/CCT subunits], confirms an active expansion of the protein folding machinery rather than passive accumulation of undegraded proteins. We also observed up-regulation of key endoplasmic reticulum-associated degradation (ERAD) components, including the AAA-ATPase CDC-48.2 [human VCP/p97] and the adaptor SEL-1 [human SEL1L]. The activation of UPR^ER^ and ERAD suggests that when lysosomal degradation is compromised, cells compensatorily activate ER quality control mechanisms to maintain ER homeostasis.

### 2.4. Systemic Upregulation of the Ubiquitin–Proteasome System During Lysosomal Storage

**Figure 4:**
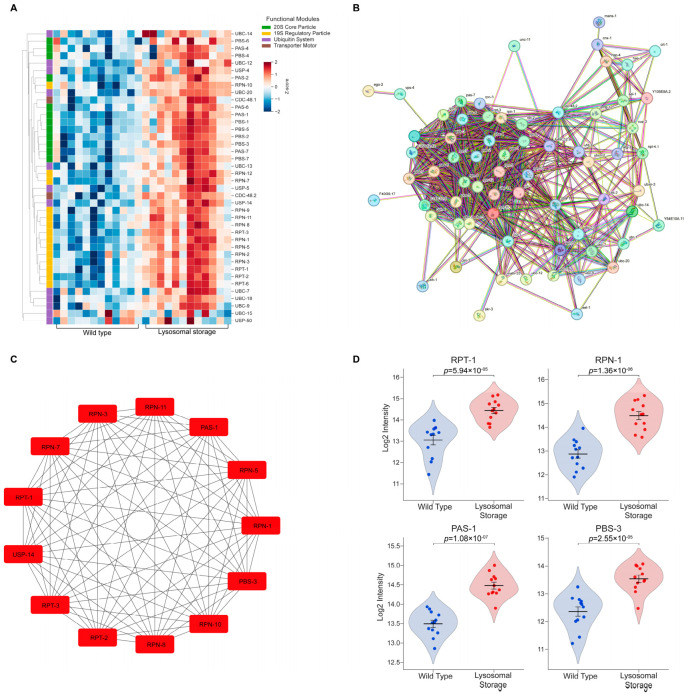
Lysosomal storage drives coordinated upregulation of the ubiquitin–proteasome system (UPS). (**A**) Hierarchical clustering heatmap showing the systemic upregulation of UPS-associated DEPs in the coelomocytes of *jefIs41[nhx-2p::CPL-1::wrmScarlet]* worms (lysosomal storage condition). The right-hand color bar annotates key functional modules of the UPS. Color intensity represents Z-score-normalized protein abundance. (**B**) PPI network of upregulated UPS components illustrates the dense interconnectivity indicative of the assembly of the complete 26S proteasome in the lysosomal storage condition. (**C**) The 13 top-ranked hub proteins from the UPS network, identified via the MCC algorithm, in the coelomocytes of *jefIs41[nhx-2p::CPL-1::wrmScarlet]* worms (lysosomal storage condition). These hubs comprise essential subunits from both the 19S and 20S proteasomal particles. (**D**) Violin plots showing obvious upregulation of representative subunits from the 19S (RPT-1, RPN-1) and 20S (PAS-1, PBS-3) particles in single coelomocytes under lysosomal storage condition. Each dot represents a single-cell measurement. Horizontal lines within the plots represent the mean ± SEM. Statistical significance was determined using a two-tailed Student’s *t*-test.

The ubiquitin–proteasome system (UPS) serves as a critical defense against proteotoxicity by degrading misfolded or damaged proteins across various cellular compartments, including the cytosol, endoplasmic reticulum (ER), and mitochondria [21,22]. A detailed heatmap of upregulated UPS components revealed a striking, near-universal induction under lysosomal storage conditions (Figure 4A), consistent with our Gene Ontology (GO) analysis highlighting proteasomal processes (Figure 3A). This global upregulation included CDC-48.1 and CDC-48.2 [human VCP/p97], which extract proteins from the ER and mitochondria for cytosolic degradation. Consequently, the enhanced UPS activity signifies a systemic effort to alleviate the protein burden accumulating in the overwhelmed ER, cytosol, and mitochondria during lysosomal dysfunction.

The PPI network of upregulated UPS components displayed a densely interconnected architecture, suggesting the active assembly of complete 26S proteasome holoenzymes rather than the isolated upregulation of individual subunits (Figure 4B). Topological analysis identified 13 hub proteins comprising essential subunits from both the 19S regulatory particle (e.g., RPN and RPT series) and the 20S core particle (e.g., PAS-1 [human PSMA6], PBS-3 [human PSMB3]), thereby ensuring full capacity for substrate recognition, unfolding, and degradation [23] (Figure 4C). These results support that lysosomal storage induces the coordinated upregulation of both 19S and 20S structural modules, which may facilitate the adaptive degradation of proteins normally cleared by lysosomal pathways. Single-cell quantification confirmed significant elevation of key components, including RPT-1 [human PSMC2] and RPN-1 [human PSMD2] (essential for substrate recognition and ATP-dependent unfolding in the 19S particle) and PAS-1 and PBS-3 (vital for the proteolytic activity of the 20S core) (Figure 4D). Collectively, these data demonstrate a massive compensatory mobilization of the UPS that expands cellular degradative capacity in response to lysosomal failure.

### 2.5. Lysosomal Failure Elicits a Complex Mitochondrial Response Characterized by Upregulated Respiratory Complexes and Enhanced Quality Control Responses

**Figure 5:**
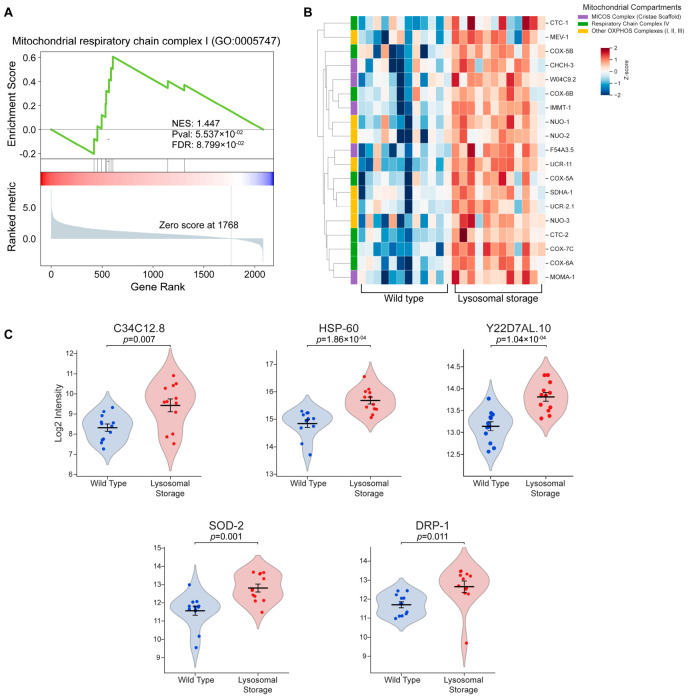
Lysosomal failure elicits a discordant mitochondrial response of compensatory bioenergetics and quality control responses. (**A**) GSEA plot showing significant enrichment of the mitochondrial respiratory chain complex I pathway (GO:0005747) in the coelomocytes of *jefIs41[nhx-2p::CPL-1::wrmScarlet]* worms (lysosomal storage condition). (**B**) Hierarchical clustering heatmap of up-regulated mitochondrial proteins in the lysosomal storage condition. The color bar on the right annotates key functional groups, including OXPHOS complexes and the MICOS complex. Color intensity represents Z-score normalized abundance. (**C**) Violin plots showing robust single-cell induction of key markers of mitochondrial protein import (C34C12.8), proteostatic stress (HSP-60, Y22D7AL.10), oxidative stress (SOD-2), and fragmentation (DRP-1) in the coelomocytes of *jefIs41[nhx-2p::CPL-1::wrmScarlet]* worms (lysosomal storage condition). Each dot represents a single cell measurement. Horizontal lines within the plots represent the mean ± SEM. Statistical significance was determined using a two-tailed Student’s *t*-test.

Beyond degradation, lysosomes recycle macromolecules into biosynthetic precursors [5,24]. We hypothesized that severe lysosomal storage would disrupt this recycling and, concomitant with the activation of other processes described above, create conditions necessitating mitochondrial remodeling. Consistent with this hypothesis, Gene Set Enrichment Analysis (GSEA) revealed significant upregulation of mitochondrial respiratory chain complex I (Figure 5A), indicating a compensatory drive for energy production.

To further characterize this bioenergetic response, we performed a targeted heatmap analysis (Figure 5B). Notably, these results aligned with our initial Gene Ontology (GO) prediction (Figure 3A), which highlighted the pronounced upregulation of specific subunits within respiratory chain complex IV. The heatmap demonstrated a robust, concurrent induction of complex IV alongside other oxidative phosphorylation (OXPHOS) subunits, including complexes I, II, and III, as well as the mitochondrial contact site and cristae organizing system (MICOS). The coordinated induction of the MICOS complex suggests active structural remodeling aimed at increasing cristae density and maximizing inner membrane surface area, representing an adaptive response to enhance respiratory efficiency during lysosomal decline [25]. The assembly of these massive multiprotein complexes requires an extensive influx of nuclear-encoded proteins into the mitochondria. Aligning with this logistical demand, our earlier network analysis identified C34C12.8 [human GRPEL1/2], a mitochondrial GrpE homolog and essential nucleotide exchange factor for the presequence translocase-associated motor (PAM) complex [26,27], as a core regulatory hub (Figure 3C). Single-cell quantitative profiling further confirmed the robust upregulation of C34C12.8 during lysosomal storage (Figure 5C), supporting a compensatory increase in mitochondrial protein import to boost mitochondrial function as lysosomal function declines.

This compensatory drive for energy, coupled with a diminished capacity to remove damaged mitochondria during lysosomal storage, may trigger strong mitochondrial stress responses. Indeed, we observed significant upregulation of mitochondrial stress responders (Figure 5C), including HSP-60 [human Hsp60], the canonical mitochondrial chaperonin indicative of severe matrix proteotoxic stress, and its obligate co-chaperonin Y22D7AL.10 [human Hsp10], which we previously identified as a core hub within the protein folding network (Figure 3C) [28]. Furthermore, the primary mitochondrial antioxidant enzyme SOD-2 [human SOD2] was highly enriched, reflecting an elevated requirement for antioxidant defense within the mitochondria [29]. Finally, the pronounced induction of DRP-1 [human DRP1], the master regulator of mitochondrial fission [30], suggests that the network is actively undergoing fragmentation, likely representing a compensatory attempt to segregate damaged mitochondria. Collectively, these data reveal a paradoxical mitochondrial state: an attempt to ramp up energy production occurring concurrently with profound mitochondrial stress responses, marking a critical compensatory mechanism to boost mitochondrial function and cope with lysosomal decline.

## 3. Discussion

We present the first high-resolution, single-cell proteomic map of the cellular response to lysosomal overload. Using the *C. elegans* coelomocyte model, we show that lysosomal storage is not an isolated defect but a systemic crisis that triggers a coordinated, tripartite compensatory response: massive induction of ER quality control machinery, wholesale expansion of UPS degradative capacity, and energy-seeking mitochondrial remodeling that ultimately exacerbates organelle distress. These findings establish a functional, hierarchical model of cellular survival strategies when the primary recycling center is overwhelmed.

A striking feature of our dataset is the profound asymmetry in protein regulation, with nearly 1000 proteins significantly upregulated. We acknowledge the inherent challenge in applying steady-state proteomics to professional scavenger cells: distinguishing actively translated stress-response proteins from passively accumulated, undegraded extracellular cargo (as likely seen with the 127 uniquely detected proteins in the lysosomal storage group) can be difficult at the single-protein level. However, our network and algorithmic analysis indicate that within the 999 shared upregulated proteins, this shift represents an active, orchestrated compensatory effort. The stoichiometric assembly of complete 26S proteasomes and the synchronous induction of ER chaperones hallmark a deliberate transcriptional and translational program. Our data suggest a logical hierarchy: when the primary degradative system (the lysosome) fails, the cell forcefully reallocates resources to activate other quality control responses to maintain cellular homeostasis.

Furthermore, our data reveal a discordant and ultimately self-defeating mitochondrial response. The simultaneous upregulation of OXPHOS complexes and MICOS structural components, alongside markers of severe proteostatic distress such as HSP-60 and SOD-2, suggests metabolic desperation rather than adaptation. During lysosomal storage, the failure of lysosomes to recycle macromolecules, combined with the demand to trigger other quality control processes, likely drives compensatory overdrive in mitochondrial bioenergetics to maintain ATP levels. However, this overactivation, occurring amidst impaired mitophagy (a lysosome-dependent process), leads to the rapid accumulation of damaged, ROS-producing mitochondria. The subsequent induction of the fission machinery via DRP-1 appears to attempt to segregate dysfunctional organelles that can no longer be efficiently cleared. This may create a vicious cycle of energy demand and escalating organelle damage, highlighting a desperate compensatory mechanism aimed at maintaining homeostasis during lysosomal storage.

While our study provides an unprecedentedly deep proteomic snapshot of this stress landscape, we emphasize that this single-cell atlas indicates the successful establishment of the *C. elegans* LSD model, serving as a system for investigating LSD biology with powerful genetic capacity for future inquiry. Our data predict a state of profound metabolic insufficiency and specific bioenergetic bottlenecks; however, only direct functional analysis can confirm the metabolic consequences of these protein-level changes. Future investigations integrating single-cell proteomics with simultaneous transcriptomic or, ideally, metabolomic profiling and genetic manipulation will be essential to resolve the causal links between transcriptional induction, protein turnover, and functional metabolic output. Such high-dimensional, single-cell datasets will be pivotal for moving beyond observation toward identifying actionable therapeutic targets. These targets could bypass lysosomal bottlenecks and restore metabolic homeostasis, not only in Lysosomal Storage Disorders but also across the growing spectrum of related neurodegenerative diseases where lysosomal failure is a central pathogenic feature.

## 4. Materials and Methods

### 4.1. Strains and Maintenance

We generated the *C. elegans* coelomocyte lysosomal storage model strain, MAT401 *jefIs41[nhx-2p::CPL-1::wrmScarlet]*, in our laboratory. Briefly, this transgenic strain was derived from the wild-type Bristol N2 parent strain via microinjection of the *nhx-2p::CPL-1::wrmScarlet* plasmid, followed by X-ray irradiation to induce stable chromosomal integration. Detailed characteristics and the comprehensive generation protocol for this strain are described in our previous work [13]. The wild-type Bristol N2 strain was obtained from the Caenorhabditis Genetics Center (CGC, University of Minnesota, Minneapolis, MN, USA). All nematodes were maintained on Nematode Growth Medium (NGM) plates seeded with Escherichia coli OP50 following standard protocols.

### 4.2. Proteomic Profiling

Single-Coelomocyte Isolation and Processing. We isolated individual coelomocytes using a visually guided microsampling workflow on the Celleagle single-cell manipulation system (Hangzhou HW-SinPro Co., Hangzhou, China). To release coelomocytes, we dissected 20–30 adult nematodes in 80 µL of PBS buffer on a concave glass slide. We identified target cells under 20× magnification within the dissected nematode suspension. Wild-type (N2) coelomocytes were identified under differential interference contrast (DIC) optics based on their distinct macroscopic morphology: they presented as conspicuously large, ovoid cells (approximately 10–15 µm in diameter), which allowed them to be visually distinguished from surrounding tissue debris. For the lysosomal storage coelomocytes (from the MAT401 strain), while the same baseline morphological criteria were applied, target identification was further confirmed by the intense red fluorescence (CPL-1::wrmScarlet) emitted from their lysosomal compartments. A fused-silica capillary probe (35 µm tip I.D.) aspirated single target cells with an aspirating volume of 2–5 nL; after a wash step, we deposited each cell into a micro-insert tube with a conical bottom containing 200 nL of reagents. To control for background contamination, we collected procedural blanks consisting of identical fluidic manipulations in cell-free areas. Following capture, we performed proteomic sample pretreatment, including cell lysis and enzymatic digestion of proteins (2.5 h), automatically within the micro-insert tubes using reagents from a commercial kit (Single Cell Protein Processing Kit, Hangzhou HW-SinPro Co., Hangzhou, China).

LC-MS/MS Analysis. We separated peptides on a nanoElute 2 system (Bruker Daltonics, Bremen, Germany) equipped with a commercial C_18_ reversed-phase capillary column (10 cm length, 50 µm I.D., 1.7 µm particles, Hangzhou HW-SinPro Co., Hangzhou, China). Separation utilized a 21-min gradient from 3% to 40% mobile phase B (0.1% formic acid in 80% acetonitrile) at a flow rate of 150 nL/min. We acquired mass spectrometry data on a timsTOF Pro instrument (Bruker Daltonics, Bremen, Germany) operating in the data-independent acquisition with parallel accumulation serial fragmentation (diaPASEF) mode, which integrates Trapped Ion Mobility Spectrometry (TIMS) for enhanced selectivity and sensitivity. Key parameters included a capillary voltage of 1750 V and a mass range of 399–1124 *m*/*z*.

### 4.3. Proteomic Data Processing and Bioinformatics Analysis

Spectral Library Generation and Protein Quantification. We processed raw data files using DIA-NN (v1.8.1) in library-free mode to generate a project-specific spectral library [31]. Spectra were searched against the UniProt *C. elegans* proteome database with trypsin specificity (maximum one missed cleavage), carbamidomethylation of cysteine as a fixed modification, and N-terminal acetylation and methionine oxidation as variable modifications. We applied a strict false discovery rate (FDR) of <1% at both the precursor and protein group levels.

Data Pre-processing, Normalization, and Quality Control. We performed downstream analysis in a Python environment (v3.8.10). We excluded samples with fewer than 900 protein identifications, retaining a total of 12 wild-type and 12 lysosomal storage single-coelomocyte proteomes for analysis. We subjected the protein quantification matrix to a rigorous filtering pipeline: first, we subtracted background noise based on procedural blanks; second, we considered proteins valid only if detected in at least four replicates within at least one biological group. We then log_2_-transformed and median-centered the filtered data. We imputed missing values using a left-censored approach based on the global data distribution (μ_imp = μ_obs − 1.8σ_obs; σ_imp = 0.3σ_obs). Finally, we applied the ComBat algorithm to remove technical variance between two acquisition batches, specifying the biological condition as a covariate to preserve true biological variation [32].

Bioinformatics Analysis and Data Visualization. We identified differentially expressed proteins (DEPs) using an FDR < 0.05 and a fold-change threshold of |FC| ≥ 1.5. We used Pearson correlation and Uniform Manifold Approximation and Projection (UMAP) for global visualization [33]. We annotated subcellular localizations using the UniProtKB database. We performed Gene Ontology (GO) enrichment analysis using goatools (v1.4.12) with a significance threshold of *p* < 0.05, sourcing annotations from the Gene Ontology Consortium [34] and WormBase [35]. We conducted Gene Set Enrichment Analysis (GSEA) to evaluate pathway-level alterations [36]; when restricted to mitochondria-related gene sets, this identified the mitochondrial respiratory chain complex I as the sole significantly enriched term (FDR < 0.25). We generated protein–protein interaction (PPI) networks using the STRING database [37] and visualized them in Cytoscape (v3.10.4), identifying hub proteins using Maximal Clique Centrality (MCC) scores from the cytoHubba plugin [38]. We generated all data visualizations in Python using the Matplotlib (v3.10.5) and Seaborn (v0.13.2) libraries. We determined statistical significance for individual protein abundance using a two-tailed Student’s *t*-test.

## Data Availability

The original contributions presented in the study are included in the article and Supplementary Materials. Further inquiries can be directed to the corresponding authors.

## Supplementary Materials

The following supporting information can be downloaded at: https://www.mdpi.com/article/10.3390/ijms27104197/s1.
